# CD133 Modulate HIF-1α Expression under Hypoxia in EMT Phenotype Pancreatic Cancer Stem-Like Cells

**DOI:** 10.3390/ijms17071025

**Published:** 2016-06-28

**Authors:** Koki Maeda, Qiang Ding, Makoto Yoshimitsu, Taisaku Kuwahata, Yumi Miyazaki, Koichirou Tsukasa, Tomomi Hayashi, Hiroyuki Shinchi, Shoji Natsugoe, Sonshin Takao

**Affiliations:** 1 Division of Cancer and Regenerative Medicine, Kagoshima University Graduate School of Medical and Dental Sciences, 8-35-1 Sakuragaoka, Kagoshima 890-8520, Japan; maeda-k@m3.kufm.kagoshima-u.ac.jp (K.M.); colleen@m3.kufm.kagoshima-u.ac.jp (Q.D.); tkuwa@m2.kufm.kagoshima-u.ac.jp (T.K.); yu-mi@m2.kufm.kagoshima-u.ac.jp (Y.M.); kt4494@m3.kufm.kagoshima-u.ac.jp (K.T.); 2 Department of Surgical Oncology and Digestive Surgery, Kagoshima University Graduate School of Medical and Dental Sciences, 8-35-1 Sakuragaoka, Kagoshima 890-8520, Japan; t-hayasi@m.kufm.kagoshima-u.ac.jp (T.H.); shinchi@m.kufm.kagoshima-u.ac.jp (H.S.); natsugoe@m2.kufm.kagoshima-u.ac.jp (S.N.); 3 Keenan Research Centre, Li Ka Shing Knowledge Institute, St. Michael’s Hospital, Tronto, ON M5T 3M7, Canada; 4 Department of Hematology and Immunology, Kagoshima University Graduate School of Medical and Dental Sciences, 8-35-1 Sakuragaoka, Kagoshima 890-8520, Japan; myoshimi@m.kufm.kagoshima-u.ac.jp; 5 Center for Innovative Therapy Research and Application, Kagoshima University Graduate School of Medical and Dental Sciences, 8-35-1 Sakuragaoka, Kagoshima 890-8520, Japan; 6 Tanegashima Medical Center, 7463 Nishi-no-omote, Nishi-no-omote 891-3198, Japan

**Keywords:** pancreatic cancer, CD133, cancer stem cell, HIF-1α, hypoxia, migration, EMT

## Abstract

Although CD133 is a known representative cancer stem cell marker, its function in tumor aggressiveness under hypoxia is not fully known. The aim of this study is to demonstrate that CD133 regulates hypoxia inducible factor (HIF)-1α expression with tumor migration. The CD133^+^ pancreatic cancer cell line, Capan1M9, was compared with the CD133^−^ cell line, shCD133M9, under hypoxia. HIF-1α expression levels were compared by Western blot, HIF-1α nucleus translocation assay and real-time (RT)-PCR. The hypoxia responsive element (HRE) was observed by luciferase assay. The migration ability was analyzed by migration and wound healing assays. Epithelial mesenchymal transition (EMT) related genes were analyzed by real-time RT-PCR. HIF-1α was highly expressed in Capan1M9 compared to shCD133M9 under hypoxia because of the high activation of HRE. Furthermore, the migration ability of Capan1M9 was higher than that of shCD133M9 under hypoxia, suggesting higher expression of EMT related genes in Capan1M9 compared to shCD133M9. Conclusion: HIF-1α expression under hypoxia in CD133^+^ pancreatic cancer cells correlated with tumor cell migration through EMT gene expression. Understanding the function of CD133 in cancer aggressiveness provides a novel therapeutic approach to eradicate pancreatic cancer stem cells.

## 1. Introduction

Pancreatic cancer is a highly lethal disease that is usually diagnosed at an advanced stage and is relatively insensitive to any current therapy. A potential reason for the failure of the classical therapeutic approach might be explained by cancer stem cell theory [1,2]. In pancreatic cancer, cancer stem cells (CSCs) were isolated by using cell surface markers, such as CD133^+^ [3], CD44^+^CD24^+^ESA^+^ [4] or c-Met^+^ [5]. 

We previously reported that CD133 expression in pancreatic cancer is correlated with poor prognosis, lymph node metastasis and micro-vessel density [6]. However, there is a lack of evidence regarding how this cell surface marker modulates the intracellular stemness properties or tumorigenicity of pancreatic cancer. Some reports showed a possibility of a direct relationship between CD133 and Src resulting in epithelial mesenchymal transition (EMT) and stemness properties [7]. Recently, we found that the CD133/Src/slug signaling axis correlates to *N*-cadherin expression in pancreatic cancer [8].

On the other hand, the hypoxic microenvironment is common in pancreatic cancer because of its insufficient blood supply with desmoplastic morphology [9]. A number of epidemiological and clinical studies have suggested that the hypoxia and hypoxia-induced signaling pathway are highly associated with the poor clinical outcome of patients diagnosed with many solid tumors. In particular, hypoxia inducible factor (HIF) plays a major role in cancer cell survival and aggressiveness in hypoxia [10]. Clinicopathological evidence showed negative correlation between patient survival and HIF-1α expression [11,12].

Several reports demonstrated that hypoxia increased a percentage of CD133 expression in cancer cell lines [13,14]. Notably, CD133^+^ cells under hypoxia showed high self-renewal potential in glioblastoma [13], glioma [15] and high invasion and migration ability in pancreatic cancer cell line [14]. CD133-overexpressing head and neck squamous cell carcinoma enhanced the expression of HIF-1α and HIF-2α [7].

In the present study, we demonstrated that CD133 expression in pancreatic cancer plays a role in initiating HIF-1α expression through increasing transcriptional activity under hypoxia following encouraged tumor migration ability and EMT phenomenon.

## 2. Results

### 2.1. CD133^+^ Cells Obtained Tolerance to Hypoxia

**Figure 1:**
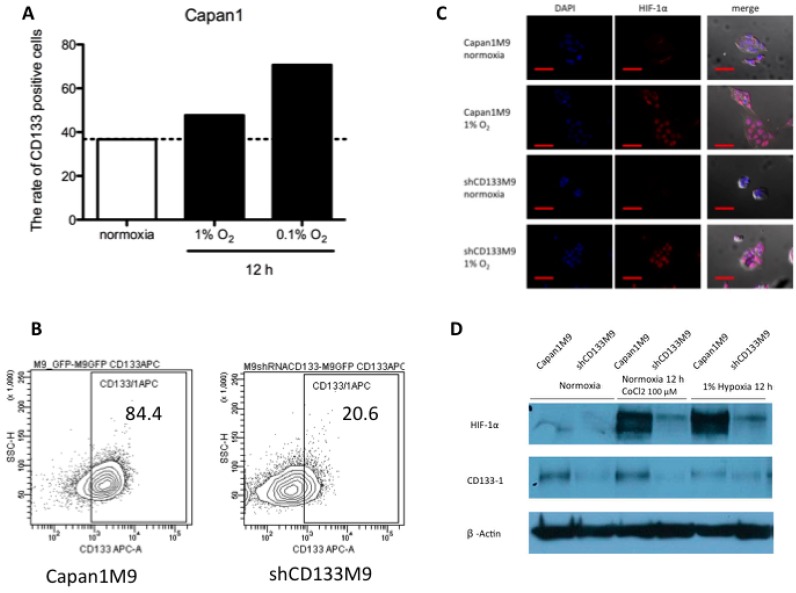
CD133^+^ cells obtained tolerance to hypoxia. (**A**) Flow cytometric analysis showed an increase of the rate of CD133^+^ cells under 1% hypoxia and 0.1% hypoxia; (**B**) Capan1M9 cells showed 84.4% CD133 expression and shCD133M9 cells showed only 20.6% CD133 positive rate by flow cytometric analysis; (**C**) Fluorescent immunostaining showed that hypoxia inducible factor (HIF)-1α expressed in nuclei of Capan1M9 and shCD133M9 under hypoxia. Scale bars show 50 mm as a red bar; (**D**) Western blot analysis proved RNAi methods effectively down-regulated CD133 expression and the difference of HIF-1α expression level.

Capan-1 cells were incubated under 1% hypoxia and 0.1% hypoxia for 12 h and CD133 expression ratio was analyzed flow cytometry. The percentage of CD133 cells increased under each hypoxic condition (Figure 1A). Thus, CD133^+^ cells tended to obtain tolerance to hypoxia. To examine the possibility that CD133 affects HIF-1α protein levels to modulate cellular responses to hypoxia, we used CD133^+^ cells (Capan1M9) and CD133^−^ cells (shCD133M9) (Figure 1B). Flow cytometry analysis showed that CD133^+^ cells were 84.4% Capan1M9 and 20.6% shCD133M9 cells. HIF-1α was expressed in the nuclei of Capan1M9 and shCD133M9 cells under hypoxia (Figure 1C). Under hyoxia incubation or hypoxic mimetic agent, CoCl_2_ treatment, HIF-1α expression was increased in CD133M9 cells. In contrast, HIF-1α expression did not increase with CD133 knockdown in shCE133M9 cells (Figure 1D). These results indicated that CD133 expression is involved in regulating the expression of HIF-1α under hypoxia.

### 2.2. CD133 Positively Regulates HIF-1α under Hypoxia

**Figure 2:**
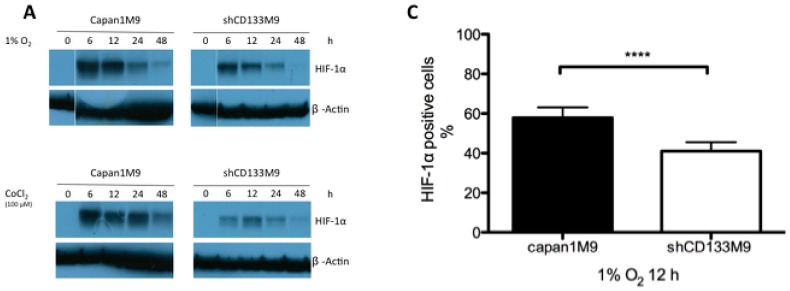

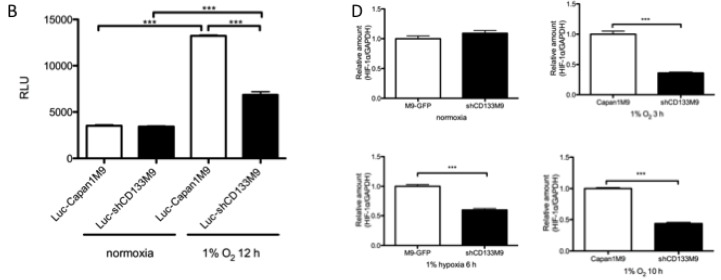
CD133 regulates HIF-1α under hypoxic condition. (**A**) Western blots confirmed CD133 silencing decreased HIF-1α protein levels in each period, both under 1% hypoxia and normoxia with CoCl_2_ administration; (**B**) In Capan1M9 and shCD133M9 transfected with HIF-1α reporter plasmid, there are significant difference of HIF-1α transactivating activity. CD133 silencing also decreased HIF-1α transactivation activity; (**C**) The ratio of HIF-1α^+^ cells was counted after 12 h 1% O_2_ exposure by HIF-1α nuclear translocation assay. HIF-1α^+^ cells of shCD133M9 were smaller than that of Capan1M9. (**D**) RT-PCR confirmed CD133 silencing decreased HIF-1α mRNA levels in each hypoxic period. Data represent mean ± SD of three experiments; *** *p* < 0.001; **** *p* < 0.0001.

HIF-1α protein levels were markedly decreased in shCD133M9 cells along with the hypoxia incubation, but not in Capan1M9 cells under hypoxia. Similar results were obtained when cells were treated with the hypoxia-mimetic agent CoCl_2_ (Figure 2A). It was also observed that CD133 downregulation reduced the translocation of HIF-1α to the nuclei in the HIF-1α nuclear translocation assay. The effect of CD133 downregulation was examined with use of ArrayScan VTI HCS Reader (Thermo Fisher Scientific, Waltham, MA, USA). The percentage of HIF-1α^+^ cells in shCD133M9 cells was significantly smaller than that of Capan1M9 under hypoxia (Figure 2B). We next examined the difference of HIF-1α transcriptional activity under hypoxia, Capan1M9 and shCD133M9 cells were transfected with HIF-1α reporter plasmid. CD133 knockdown significantly decreased the HIF-1α transcriptional activity under hypoxia observed in a Luciferase reporter assay (Figure 2C). These results were similar and supportive of the conclusion based on Western blot analysis (Figure 2A). A significant difference was observed in HIF-1α mRNA levels between Capan1M9 and shCD133M9 cells only under hypoxia (Figure 2D). Thus, CD133 positively regulated HIF-1α mRNA and protein level, and consequently upregulated the HIF-1α target genes reactivation under hypoxia.

### 2.3. CD133 Plays an Important Role in Tumor Cell Migration under Hypoxia

**Figure 3:**
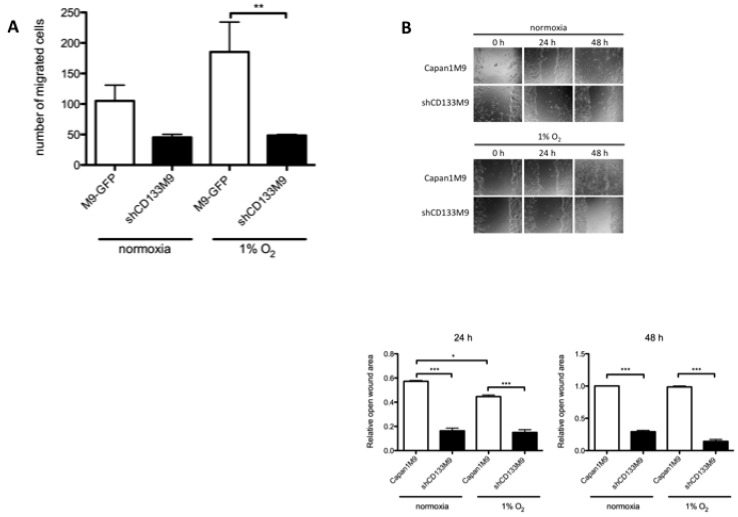
CD133 plays an important role in pancreatic cancer cell migration under hypoxia. (**A**) Boyden chamber migration assay showed CD133 silencing reduced the migratory ability of Capan1M9 both in normoxia and hypoxia. Capan1M9 only enhanced its migration ability under hypoxia; (**B**) Wound healing assay also showed CD133 silencing reduced the migratory ability of Capan1M9 in normoxia and hypoxia. Hypoxia discouraged wound healing of Capan1M9 and shCD133M9. Wide error bars for Capan1M9 cell migrations are due to varying cell number between experiments. Data represent mean ± SD of three experiments; * *p* < 0.05; ** *p* < 0.01; *** *p* < 0.001.

HIF-1α plays a major role in tumor migration under hypoxia [16]. To study the CD133 knockdown effect to tumor migration, we performed a migration assay and a wound healing assay. The number of migrated cells increased in Capan1M9 cells under hypoxia but not in shCD133M9 cells. Compared to the migration numbers in normoxia, only Capan1M9 cells showed an increase in migration (Figure 3A). Wound healing assay also showed that Capan1M9 had more migration ability than shCD133M9 cells under normoxia and hypoxia, although even Capan1M9 decreased wound healing ability under hypoxia compared to normoxia (Figure 3B). These results indicated that CD133 played an important role in pancreatic cancer cell tumor migration, especially under hypoxia.

### 2.4. CD133 Contributes to Epithelial Mesenchymal Transition (EMT) Phenomenon under Hypoxia

**Figure 4:**
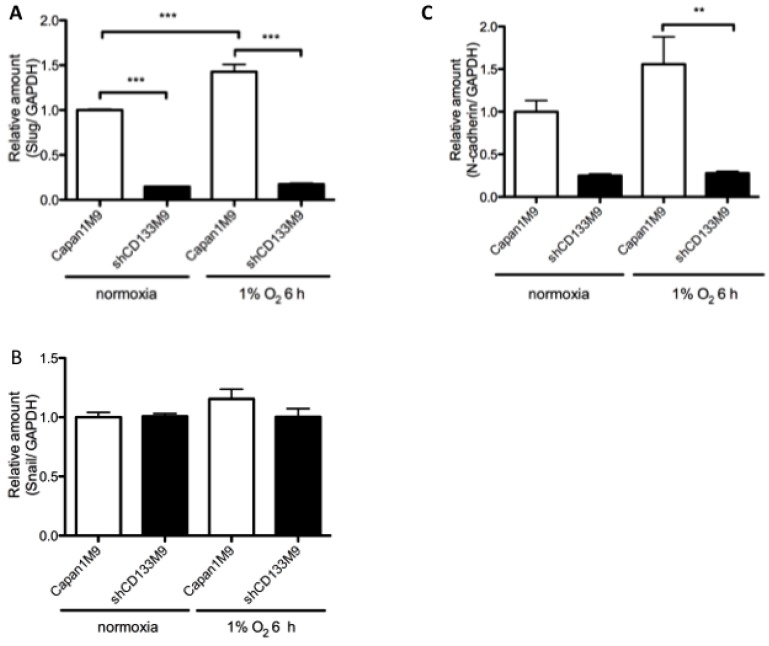
CD133 contributes to EMT phenomenon under hypoxia. (**A**) CD133 silencing down-regulated Slug mRNA level significantly. Hypoxia gained the Slug mRNA level only in Capan1M9; (**B**) Significant difference was not shown in Snail mRNA level; (**C**) CD133 silencing down-regulated *N*-cadherin mRNA level. Data represent mean ± SD of three experiments; ** *p* < 0.01; *** *p* < 0.001.

The increased metastatic potential of hypoxic cancers might result from morphological changes in cell biology called EMT. In the present study, DNA microarray analysis showed the different expression of Slug (Snail2) and *N*-cadherin (CDH2) between Capan1M9 and shCD133M9 cells under normoxia (Table S1 and Figure S1). We then compared these genes present within Capan1M9 and shCD133M9 cells under hypoxia by real-time RT-PCR. The *N*-cadherin mRNA level in Capan1M9 cells was significantly lower than that in shCD133M9 cells. The Slug mRNA level in Capan1M9 cells was significantly higher than that in shCD133M9 cells. These results suggested that CD133 may also contribute to EMT phenomenon under hypoxia (Figure 4).

## 3. Discussion

In the present study, we investigated the roles of CD133 in tumor migration via encouraging HIF-1α expression under hypoxia. The identification of the CD133/HIF-1α signaling axis supposes that CD133 is a functional marker of pancreatic CSCs, providing theoretical support for the utility of CD133 in defining pancreatic CSCs. Not all pancreatic cancer cell lines are known to have a relatively high expression of CD133 [17]. Therefore, we choose Capan1M9 in which CD133 was enriched by 9 times migration assay in this article [18].

The finding that the percentage of CD133 in the pancreatic cancer cell line increased under hypoxia suggests that CD133 expression contributes to tumor survival under hypoxia. Interestingly, Hashimoto et al. [14] demonstrated that other pancreatic cancer cell lines also gained CD133 expression by hypoxic stimulation. Several metabolic stressors including hypoxia, low pH, glucose level and molecules such as β_2_-Adrenogenic signaling and pyruvate kinase M2 (PKM2), induce HIF-1α expression results in tumor survival [19]. Moreover, a hypoxic environment is required for cancer stem cell function [20]. This suggests that hypoxia and the HIF signaling pathway lead to enrichment of CSCs. Li et al. [13] showed that HIF-2α was induced during hypoxia and was critical for the tumorigenicity of glioma stem cells. On the other hand, Yin et al. [21] proved that HIF-1α is essential to maintain CSCs of haematological malignancies. Therefore we focus on the relationship between CD133 and HIF-1α.

In a human pancreatic cancer cell line, we found that the downregulation of CD133 expression resulted in a decrease in HIF-1α expression, suggesting that CD133 has some role in influencing HIF-1α expression. CD133 is correlated with poor prognosis, lymph node metastasis and micro vessel density in pancreatic cancer [6]. Further, we recently reported that HIF-1α expression levels in pancreatic cancer are also associated with tumor progression, fibrotic focus, angiogenesis, cell migration, cell invasion and hepatic metastasis [11]. Soeda et al. [15] showed that hypoxia could promote CD133^+^ cancer stem-like cells expansion by upregulating HIF-1α. Furthermore, Hashimoto et al. [14] also found that hypoxia could encourage CD133 expression with HIF-1α. However, the mechanism of an interaction between CD133 and HIF-1α is still unclear. In the present study, we showed the possibility that CD133 affects HIF-1α expression and migration in pancreatic cancer.

CD133^+^ cells (Capan1M9) showed high expression of HIF-1α compared to CD133^−^ cells (shCD133M9) not only under hypoxia but also under normoxia with CoCl_2_ administration (Figure 2A). CoCl_2_ is known as a hypoxic mimicking agent, which blocks HIF-1α degradation [22,23]. This result suggested that CD133 enhances HIF-1α expression by increasing HIF-1α mRNA transcription or translation without inhibiting HIF-1α degradation. CoCl_2_, hypoxia mimicking agent occupies the VHL-binding domain of HIF-1α and prevents the degradation of HIF-1α [23]. Dominant HIF-1α protein expression in Capan1M9 compared with in shCD133M9 caused by HIF-1α mRNA level difference. In Figure 2D, CD133 silencing decreased HIF-1α mRNA levels in each hypoxic period. Therefore, canonical HIF degradation pathway’s participation in these experiments seems limited. The HIF-1α nucleus translocation assay’s results also supported the idea that CD133 knockdown induced a decrease of HIF-1α expression under hypoxia (Figure 2C).

Mechanisms regarding how CD133 controls HIF-1α expression are not fully known although several possible explanatory mechanisms exist. First, CD133 regulates HIF-1α through an oncogenic pathway. Increasing evidence suggests the functional association of CD133^+^ CSCs with Akt signaling. CD133^+^ tumor cells derived from hepatoma, colon cancer, and neuroblastoma consistently displayed increased phosphor-Akt levels compared with matched CD133^−^ tumor cells [24,25,26]. There are several mechanisms controlling HIF through transcripitonal and post-transcriptional regulation [27]. CD133 encourages NF-κB activation in pancreatic cancer cell line [28]. Moreover, hypoxia and oxidative stress elevates HIF-1α mRNA levels transcriptionally through NF-κB activation involving a PI3K-dependent pathway [29]. It implied that a CD133-NF-κB-HIF axis would be the mechanism under hypoxia to regulate HIF-1a mRNA expression. Second, CD133 directly stimulates HIF-1α expression. Our data showed that CD133 was detected not only in the cytosol but also in the nucleus of Capan1M9 cells (Figure S2), and we used antibodies directed against AC133, an extracellular epitope of CD133 in Western blot analysis [30]. This result suggested that CD133 itself would possibly interact not only with cytosol organisms but also intranuclear organisms directly.

CD133 knockdown decreased cell migration under normoxia and hypoxia in migration assay (Figure 3A). We previously reported that CD133 knockdown reduced migration capacity in pancreatic cancer cells [8]. In addition to slug participation to migration, the decrease of migration ability induced by CD133 knockdown was much more obvious in hypoxia than in normoxia, because of the difference of HIF-1α expression levels. On the other hand, even though Capan1M9 demonstrated higher migration ability than shCD133M9 in normoxia and hypoxia, hypoxia reduced wound-healing speed both in Capan1M9 and shCD133M9. This may be explained by the wound healing assay’s results reflecting a combined cell ability of proliferation and migration [31]. 

CSC theory has been widely accepted as a central principle to explain tumor aggressiveness, recurrence, chemoresistance and even metastasis through EMT phenomenon [2,32,33,34]. We assume that pancreatic CSCs characterized CD133 expression may have a survival advantage under unfavorable hypoxic conditions, since EMT has been demonstrated to contribute to drug resistance in pancreatic cancer [32,35]. In addition, a previous investigator reported that only pancreatic cancer cells with CSC characteristics acquired high migratory potential with hypoxia induced EMT [36]. We showed that CD133 regulates Slug and *N*-cadherin via the Src pathway [8]. The connection between HIF and Slug in pancreatic cancer is not very clear. However, Alexei et al. [36] showed HIF and slug co-expression in patient tumor tissue of pancreatic cancer by fluorescent immunostaining. Even under hypoxia, CD133^+^ cells showed high Slug mRNA level and *N*-cadherin levels compared with CD133^−^ cells (Figure 4). Therefore, an additional use of HIF-1α inhibitor in combination with conventional therapy is expected to help overcome CD133^+^ CSCs [37].

## 4. Experimental Section

### 4.1. Cell Culture

Human pancreatic cancer cell line, Capan-1, was purchased from the American Type Culture Collection (ATCC, Manassas, VA, USA) and cultured in DMEM/F12 (Sigma, St. Louis, MO, USA) medium containing 10% fetal bovine serum (FBS; Invitrogen, Carlsbad, CA, USA) supplemented with 100 units/mL penicillin and 100 mg/mL streptomycin. For normoxic conditions, cells were cultured in 5% CO_2_ and 95% air at 37 °C under a humidified atmosphere. For hypoxic conditions, cells were cultured in 1% O_2_, 5% CO_2_ and 94% N_2_ at 37 °C under a humidified atmosphere using a multigas incubator. Extremely hypoxic (0.1% O_2_) conditions were generated with AnaeroPack (Mitsubishi Gas Chemical Co., Tokyo, Japan).

### 4.2. Establishing Capan1M9 Cells

To examine the function of CD133, we developed novel cell line Capan1M9 and shCD133 M9 from Capan1 [18]. CD133—cells named shCD133M9 were established from Capan1M9 by using the RNAi method.

### 4.3. Fluorescent Immunostaining

Cells (5 × 10^4^/mL/well) were seeded on glass coverslips and incubated in normoxia or 1% O_2_ hypoxia. After incubation for 20 h, cells were fixed with 4% formaldehyde in Phosphate buffered saline (PBS) for 10 min at room temperature. Cells were incubated with 50 mM NH_4_Cl for 10 min at room temperature after washed and permeabilized with 0.2% Triton X-100. A primary antibody of mouse anti-HIF-1α (BD Bioscience, San Jose, CA, USA) were incubated with the appropriate dilutions 1:200 in PBS with BSA overnight at 4 °C. Slides were washed and cells were incubated with secondary antibody at 1:800 dilutions for 1 h at room temperature. After more washes, nuclei were labelled with DAPI imaged with a fluorescence microscope (Berlin, Germany).

### 4.4. HIF-1α Nuclear Translocation Assay

Cells (5 × 10^4^) were seeded on glass coverslips and incubated in normoxia or 1% O_2_ hypoxia. After 12 h incubation, cells were stained for HIF-1α nuclear translocation assay using the same methods as that of fluorescent immunostaining. HIF-1α translocation into nuclei were detected using an ArrayScan VTI HCS Reader (Thermo Fisher Scientific). The percentage of HIF-1α^+^ cells was calculated in 500 cells selected at random.

### 4.5. Cell Lysis, Nuclear Protein Extraction and Immunoblotting

Whole-cell extraction was performed with 1× laemmli sample buffer (Bio-Rad, Hercules, CA, USA) with 1% 2-Mercaptoethanol and lysates were boiled for 5 min and then clarified by centrifugation at 15,000× *g* for 15 min. For protein extraction, a Nuclear/Cytosol Fraction Kit (BioVision, Milpitas, CA, USA) was used according to the manufacturer’s protocol. Protein concentration was determined with a Pierce Microplate BCA Protein Assay Kit-Reducing Agent Compatible (Pierce Biotechnology, Rockford, IL, USA), and whole-cell extract lysate (50 µg) was separated by electrophoresis on sodium dodecyl sulfate (SDS)-polyacrylamide gel, and transferred to nitrocellulose membranes. The membranes were incubated with a 1:100–200 dilution of the human polyclonal or monoclonal antibodies: HIF-1α (Becton Dickinson, Franklin Lakes, NJ, USA), β-actin Sigma, St. Louis, MO, USA), CD133 (Miltenyi Biotec, Cologne, Germany) followed by a peroxidase-conjugated anti-mouse IgG antibody for the secondary reaction. As an internal control for the amount of protein loaded, β-actin was detected by use of a specific antibody. The immunocomplex was visualized by use of the ECL Western blot detection system (Amersham, Buckinghamshire, UK).

### 4.6. Migration Assay

A 24-well Cell Culture insert (8 µm pore size, Becton Dickinson) was used as the upper chamber to study effects of hypoxia on the migration ability of tumor cells (5 × 10^4^ cells/well). A suspension of tumor cells in 500 µL serum-free DMEM-F12 was added to the upper chambers, whereas the lower chambers were each filled with 500 µL chemoattractant medium (DMEM-F12 plus 10% FBS). The cells incubated for 20 h. The cells that did not invade into the membrane were removed from the inserts with a cotton swab. The cells invaded into the lower surface of membrane were fixed with 4% formalin for 10 min, stained with Hematoxylin solution for 20 min and counted under a light microscope in five parts at random.

### 4.7. Wound Healing Assay 

The CytoSelect 24-Well Wound Healing Assay (Cell Biolabs, San Diego, CA, USA) was used to analyze migration of Capan1M9 and shCD133M9 in normoxia and hypoxia. Capan1M9 cells and shCD133M9 cells were cultured onto 24-well plates that contained inserts to defined scratch areas for 24–48 h until a monolayer formed. The inserts were removed to generate a ‘wound field’. The cells were then monitored under microscope to examine migration into the wound field by original magnification (50×). The wound healing area was calculated using the software Image J (NIH, Washington, DC, USA). Migration was subsequently defined as ratio of open scratch area after 24 h and initial scratch area, 48 h and initial scratch area.

### 4.8. RNA Isolation and cDNA Synthesis

The total RNA from the cultured cells was isolated using a Qiagen RNeasy Mini kit (Qiagen, Valencia, CA, USA) according to the manufacturer’s protocols. RNA (2 µg) was reverse-transcribed using an Advantage RT-for-PCR Kit (Clontech Laboratories, Mountain View, CA, USA).

### 4.9. Quantitative Real-Time PCR

Expression levels of HIF-1α, *N*-cadherin, Snail and Slug were determined by real-time PCR using Rotor-Gene 3000 (Corbett Research, Cambridge, UK) according to the manufacturer’s instructions. The expression levels were measured using a SYBR Green I kit (Takara, Osaka, Japan). Human *GAPDH* was used for normalization. The standard curve method was used to determine expression levels of target genes. Primer sequences used are mentioned in Table S2.

### 4.10. Luciferase Reporter Assay

Capan1M9 and shCD133M9 cells expressing HRE-dependent luciferase reporter construct were established with Cignal Lenti Reporter (SABioscience, Frederick, MD, USA) according to the manufacturer’s instructions. The consensus sequence of HRE was 5′-TACGTGCT-3′ from erythropoietin genes. Cells stably expressing the HRE-reporter gene were selected with puromycin. The cells were incubated for 12 h under normoxic and hypoxic condition. The luciferase assay was performed using a Luciferase Assay System (Promega, Madison, WI, USA) according to the manufacturer’s instruction.

### 4.11. Flow Cytometric Analysis

A total of 10^6^ cells were suspended in 100 µL PBS containing 0.5% BSA. The mouse anti-human CD133 mAb (allophycocyanin (APC)-conjugated, Miltenyi Biotec) was appropriately diluted in the FcR blocking reagent to a final volume of 20 µL. The antibody was added to the cells, and mixture was incubated on ice. The cells were washed and resuspended in a suitable amount of buffer for analysis by flow cytometry. Flow cytometric analyses were carried out with a FACSAria flow cytometer (Becton Dickinson). Dead cells were excluded by 7-amino-actinomycin-D (BD Pharmingen, San Diego, CA, USA) staining.

### 4.12. Statistical Analysis

Data are presented as mean ± SEM. Statistical analysis was performed using GraphPad Prism (Graphpad Software, La Jolla, CA, USA). *p* Values < 0.05 were considered statistically significant.

## 5. Conclusions

**Figure 5:**
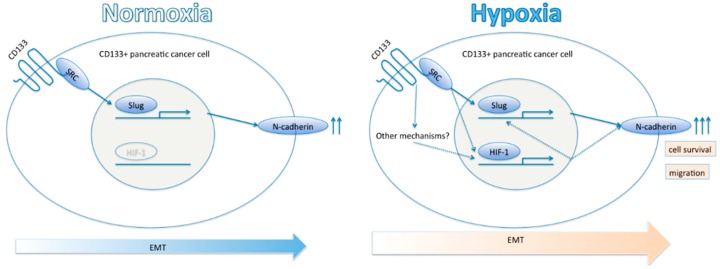
Proposed model for CD133-HIF-1α axis in pancreatic cancer stem cells. CD133 positively stimulates HIF-1α expression under hypoxia, and subsequent up-regulation of *N*-cadherin results in epithelial mesenchymal transition (EMT) phenomenon and tumor migration. Two and three arrows represent N-cadherin up-regulation.

We showed the possibility that CD133 affects HIF-1α expression, migration and EMT phenomenon in pancreatic cancer (Figure 5). A recent article suggests that over 10 years are needed to accumulate sufficient genetic change for a pancreatic cancer revolution [38]. Thus, CD133^+^ CSCs could tolerate tumor hypoxia and accumulate genetic changes for long periods. Metastasis is a decisive factor for prognosis in pancreatic cancer patients and CD133^+^ CSCs may have a key role in initiating metastasis in hypoxic cancer lesions. Therefore, detailed mechanisms of the interaction between CD133 and HIF-1α should be explored further.

## Supplementary Materials

Supplementary materials can be found at http://www.mdpi.com/1422-0067/17/7/1025/s1.
