# Supplementary material for: CD133 Modulate HIF-1α Expression under Hypoxia in EMT Phenotype Pancreatic Cancer Stem-Like Cells
Source: Int J Mol Sci. 2016 Jun 28;17(7):1025. doi: 10.3390/ijms17071025 (PMC4964401; doi:10.3390/ijms17071025)
Supplement: Supplementary file 1 [file ijms-17-01025-s001.pdf]

Supplementary Materials: CD133 Modulate HIF-1α Expression under Hypoxia in EMT Phenotype Pancreatic Cancer Stem-Like Cells

Koki Maeda, Qiang Ding, Makoto Yoshimitsu, Taisaku Kuwahata, Yumi Miyazaki, Koichirou Tsukasa, Tomomi Hayashi, Hiroyuki Shinchi, Shoji Natsugoe and Sonshin Takao

Table S1. DNA microarray analysis of epithelial mesenchymal transition (EMT)-related genes. The data represent a comparison of EMT-related genes expression between Capan1M9 and shCD133M9 cells by DNA microarray.

Gene Symbol	Description	Signal		
		**M9**	**shCD133M9**	**shSlugM9**
VIM	Homo sapiens vimentin (VIM), mRNA [NM_003380]	269.405575	14.5888225	92.51209401
FN1	Homo sapiens fibronectin 1 (FN1), transcript variant 7, mRNA [NM_054034]	23.34250625	3.9644705	5.527785483
SNAI2	Homo sapiens snail homolog 2 (Drosophila) (SNAI2), mRNA [NM_003068]	56.742035	7.067408625	21.28878
CDH2	Homo sapiens cadherin 2, type 1, *N*-cadherin (neuronal) (CDH2), mRNA [NM_001792]	695.0580281	257.1971135	241.3556833
NOTCH1	Homo sapiens notch 1 (NOTCH1), mRNA [NM_017617]	675.14325	247.6606125	291.6518125
IL1RN	Homo sapiens interleukin 1 receptor antagonist (IL1RN), transcript variant 4, mRNA [NM_173843]	15554.5	4020.711	10842.87788
COL5A2	Homo sapiens collagen, type V, α 2 (COL5A2), mRNA [NM_000393]	115.58975	87.9014825	40.24735375
KRT14	Homo sapiens keratin 14 (KRT14), mRNA [NM_000526]	723.035525	327.4258219	482.6625007
MITF	Homo sapiens microphthalmia-associated transcription factor (MITF), transcript variant 1, mRNA [NM_198159]	1677.09075	554.6804375	1361.508872
ITGAV	Homo sapiens integrin, α V (vitronectin receptor, α polypeptide, antigen CD51) (ITGAV), transcript variant 1, mRNA [NM_002210]	10146.99361	4141.590421	7773.431025
TGFB2	Homo sapiens transforming growth factor, β 2 (TGFB2), transcript variant 2, mRNA [NM_003238]	654.2277412	283.6823875	505.3588487
MSN	Homo sapiens moesin (MSN), mRNA [NM_002444]	536.432725	479.7053125	186.072775
FZD7	Homo sapiens frizzled family receptor 7 (FZD7), mRNA [NM_003507]	817.834408	320.1853875	734.7740634
TMEM132A	Homo sapiens transmembrane protein 132A (TMEM132A), transcript variant 1, mRNA [NM_017870]	11660.30125	6088.394125	9303.731625
ITGB1	Homo sapiens integrin, β 1 (fibronectin receptor, β polypeptide, antigen CD29 includes MDF2, MSK12) (ITGB1), transcript variant 1E, mRNA [NM_133376]	1941.083113	1362.83509	1243.129072

Table S1. *Cont.*

Gene Symbol	Description	Signal		
		M9	shCD133M9	shSlugM9
KRT7	Homo sapiens keratin 7 (KRT7), mRNA [NM_005556]	205747.9875	108527.3875	183474.725
MMP9	Homo sapiens matrix metallopeptidase 9 (gelatinase B, 92 kDa gelatinase, 92 kDa type IV collagenase) (MMP9), mRNA [NM_004994]	181.6547384	75.3131224	183.5654288
FOXC2	Homo sapiens forkhead box C2 (MFH-1, mesenchyme forkhead 1) (FOXC2), mRNA [NM_005251]	23.70205	6.620899	27.35915625
ZEB1	Homo sapiens zinc finger E-box binding homeobox 1 (ZEB1), transcript variant 1, mRNA [NM_001128128]	26.05846125	16.5438525	25.28960125
CAV2	Homo sapiens caveolin 2 (CAV2), transcript variant 1, mRNA [NM_001233]	607.0546613	605.8412483	369.5984673
STEAP1	Homo sapiens six transmembrane epithelial antigen of the prostate 1 (STEAP1), mRNA [NM_012449]	2937.4265	1382.112792	3367.506625
TSPAN13	Homo sapiens tetraspanin 13 (TSPAN13), mRNA [NM_014399]	2238.7915	1407.64225	2330.256879
SNAI1	Homo sapiens snail homolog 1 (Drosophila) (SNAI1), mRNA [NM_005985]	128.0035525	104.0177297	117.4491913
EGFR	Homo sapiens epidermal growth factor receptor (EGFR), transcript variant 1, mRNA [NM_005228]	289.3324763	219.9080181	286.204883
GNG11	Homo sapiens guanine nucleotide binding protein (G protein), gamma 11 (GNG11), mRNA [NM_004126]	202.7561704	139.2596253	217.1940395
PLEK2	Homo sapiens pleckstrin 2 (PLEK2), mRNA [NM_016445]	5031.790125	3903.478625	5310.925875
PTK2	Homo sapiens PTK2 protein tyrosine kinase 2 (PTK2), transcript variant 1, mRNA [NM_153831]	848.3526125	502.7078875	1069.863417
ITGA5	Homo sapiens integrin, α 5 (fibronectin receptor, α polypeptide) (ITGA5), mRNA [NM_002205]	174.6968625	136.31525	190.7827375
F11R	Homo sapiens F11 receptor (F11R), mRNA [NM_016946]	28024.39125	26077.105	27275.0425
SERPINE1	Homo sapiens serpin peptidase inhibitor, clade E (nexin, plasminogen activator inhibitor type 1), member 1 (SERPINE1), transcript variant 1, mRNA [NM_000602]	13.14648905	15.30358359	10.38988336
CDH1	Homo sapiens cadherin 1, type 1, *E*-cadherin (epithelial) (CDH1), mRNA [NM_004360]	9811.239324	10611.02271	8684.681088
ILK	Homo sapiens integrin-linked kinase (ILK), transcript variant 3, mRNA [NM_001014795]	2126.26939	1718.618748	2515.711173
CAMK2N1	Homo sapiens calcium/calmodulin-dependent protein kinase II inhibitor 1 (CAMK2N1), mRNA [NM_018584]	5600.74683	4019.404653	7821.859197
FGFBP1	Homo sapiens fibroblast growth factor binding protein 1 (FGFBP1), mRNA [NM_005130]	2031.222625	3695.347	919.887475
ESR1	Homo sapiens estrogen receptor 1 (ESR1), transcript variant 1, mRNA [NM_000125]	28.5033525	40.367195	25.51461

Table S1. *Cont.*

Gene Symbol	Description	Signal		
		**M9**	**shCD133M9**	**shSlugM9**
TGFB1	Homo sapiens transforming growth factor, β 1 (TGFβ1), mRNA [NM_000660]	820.4119617	897.561245	1017.6701
OCLN	Homo sapiens occludin (OCLN), transcript variant 1, mRNA [NM_002538]	432.1801625	643.668125	367.4899
SNAI3	Homo sapiens snail homolog 3 (Drosophila) (SNAI3), mRNA [NM_178310]	86.723435	104.0595921	109.3763113
TIMP1	Homo sapiens TIMP metallopeptidase inhibitor 1 (TIMP1), mRNA [NM_003254]	3068.434625	3628.826375	4213.724439
DSP	Homo sapiens desmoplakin (DSP), transcript variant 1, mRNA [NM_004415]	3972.784375	9276.67825	2570.304472
IGFBP4	Homo sapiens insulin-like growth factor binding protein 4 (IGFBP4), mRNA [NM_001552]	446.6832875	706.3216625	666.6703875
ERBB3	Homo sapiens v-erb-b2 erythroblastic leukemia viral oncogene homolog 3 (avian) (ERBB3), transcript variant 1, mRNA [NM_001982]	3384.575438	6670.921	4453.137855
JAG1	Homo sapiens jagged 1 (JAG1), mRNA [NM_000214]	76.85549875	206.2662629	72.55606
CALD1	Homo sapiens caldesmon 1 (CALD1), transcript variant 1, mRNA [NM_033138]	146.2501125	527.5935875	71.47478
DSC2	Homo sapiens desmocollin 2 (DSC2), transcript variant Dsc2a, mRNA [NM_024422]	147.7242	724.674875	140.49815
RGS2	Homo sapiens regulator of G-protein signaling 2, 24 kDa (RGS2), mRNA [NM_002923]	308.1948151	1625.207484	332.2243848
TCF4	Homo sapiens transcription factor 4 (TCF4), transcript variant 2, mRNA [NM_003199]	34.24982982	192.5447125	25.61543875
WNT11	Homo sapiens wingless-type MMTV integration site family, member 11 (WNT11), mRNA [NM_004626]	71.56063	396.12025	94.65079553

Table S2. Primers list for real-time reverse transcriptase-polymerase chain reaction.

Genes	Primer Sets Forward (5′–3′)	Primer Sets Reverse (5′–3′)
HIF1A	CCACAGGACAGTACAGGATG	TCAAGTCGTGCTGAATAATACC
N-cadherin	CATCAACCGGCTTAATGGTG	ACTTTCACACGCAGGATGGA
Snail	TCGGAAGCCTAACTACAGCGA	AGATGAGCATTGGCAGCGAG
Slug	AAGCATTTCAACGCCTCCAAA	GGATCTCTGGTTGTGGTATGACA
GAPDH	CAGGAGCGAGATCCCT	GGTGCTAAGCAGTTGGT

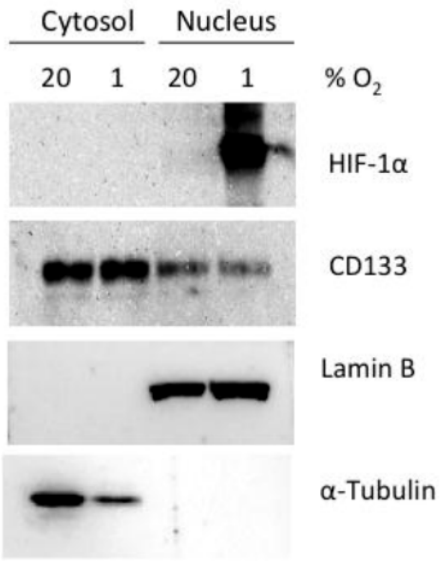

Figure S1. CD133 expression both in nucleus and cytosol. CD133 expresses both in nucleus and cytosol. Western blot assays of HIF-1α, CD133, Lamin B and α-tubulin protein in nuclear and cytosolic lysates prepared from Capan1M9 cells exposed to 20% or 1% O_2 for 6 h. Anti-CD133 antibody directed against AC133, an extracellular epitope of CD133 was detected not only in cytosol but also in nucleus. This result suggests CD133 might exist in nucleus.

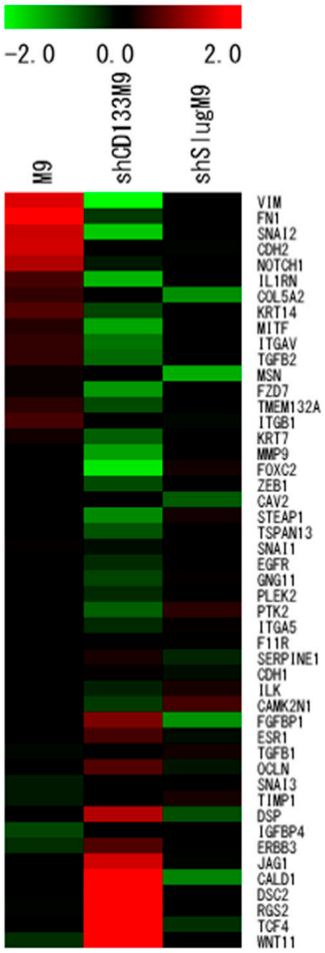

Figure S2. A heatmap of the DNA microarray demonstrates the distinct molecular profiles of Capan1M9 and shCD133M9 cells.
